# Fumigant Toxicity in *Myzus persicae* Sulzer (Hemiptera: Aphididae): Controlled Release of *(E)*-anethole from Microspheres

**DOI:** 10.3390/plants9010124

**Published:** 2020-01-18

**Authors:** María J. Pascual-Villalobos, Manuel Cantó-Tejero, Pedro Guirao, María D. López

**Affiliations:** 1 Instituto Murciano de Investigación y Desarrollo Agrario y Alimentario (IMIDA), C/Mayor S/N La Alberca, 30150 Murcia, Spain; manuel.canto@carm.es; 2 Departamento de Producción Vegetal y Microbiología, Universidad Miguel Hernández, Escuela Politécnica Superior de Orihuela, Carretera de Beniel Km. 3.2, 03312 Orihuela, Alicante, Spain; pedro.guirao@umh.es; 3 Departamento de Producción Vegetal, Facultad de Agronomía, Universidad de Concepción, Campus Chillán, Avenida Vicente Méndez 595, P.O. Box 537, Chillán 3812120, Chile; lolalopezbelchi@gmail.com

**Keywords:** encapsulation, essential oils, botanical active substances, insecticidal activity, aphids, anise, fennel, oil emulsion entrapment, spray drying

## Abstract

*(E)*-anethole is a phenylpropanoid that is the main compound found in the essential oils (EOs) of anise and fennel seeds, and either fumigant or direct contact activity of this compound has been demonstrated against aphids and stored product pests. In this work, solid microspheres were prepared by three methods—oil emulsion entrapment, spray-drying, and complexed with β-cyclodextrin. Fumigation activity of each microsphere preparation was tested against the green peach aphid, *Myzus persicae* Sulzer (Hemiptera: Aphididae), on pepper leaves. The best insecticidal activity was with *(E)*-anethole encapsulated in oil emulsion beads and introduced to aphids as a vapour over 24 h, with an LC_50_ of 0.415 μL/L compared to 0.336 μL/L of vapors from free *(E)*-anethole. Scanning electron microscopy of the beads revealed a compact surface with low porosity that produced a controlled release of the bioactive for more than 21 d, whilst most of the volatile was evaporated within two days if applied unformulated. Spray drying gave spherical particles with the greatest encapsulated yield (73%) of 6.15 g of *(E)*-anethole incorporated per 100 g of powder. Further work will be done on improving the formulation methods and testing the solid microspheres in all aphid stages scaling up the experimental assay. It is foreseen that nanotechnology will play a role in future developments of low risk plant protection products.

## 1. Introduction

*(E)*-anethole [trans-1-methoxy-4-(C1-propenyl) benzene] is an aromatic ether synthesized by some plants. This phenylpropanoid is the main compound in the essential oil of umbelifers such as anise (*Pimpinella anisum* L.) or fennel (*Foeniculum vulgare* Miller) but it is also present in other plant families-Schisandraceae-*Illicium verum* Hook. f, -Rutaceae-*Clausena anisata* (Willd) Hook f ex Benth,-Backhousiaceae-*Backhousia anisata* Vickery and -Magnoliaceae-*Magnolia salicifolia* (Sieb et Zucc) Maxim. [1,2,3,4,5].

The Apiaceae (formerly Umbelliferae) family comprises vegetables (celery-*Apium graveolens* L., parsley-*Petroselinum sativum* L., coriander-*Coriandrum sativum* L.), herbs, and spices (anise, fennel, cumin-*Cuminum cyminum* L.). Aniseeds have long been used to make schnapps like the popular French pastis, a beverage distilled from anise, liquorice, and fennel seeds macerate.

Fumigant toxicity of anise and cumin essential oils has been reported against the cotton aphid (*Aphis gossypii* Glover (Hemiptera: Aphididae) [6]. Vapours of anise essential oil (EO) or its main compound *(E)*-anethole were toxic (LD_90_ = 0.18 µL/cm^2^ or 0.14 µL/cm^2^ respectively) to the bird cherry-oat aphid (*Rhopalosiphum padi* L., Hemiptera: Aphididae) in a laboratory bioassay within small air-tight dishes (2.2 × 2.2 × 1 cm^3^), according to reference [1].

A blend of *(E)*-anethole, limonene, and fenchone at 880 ppm was toxic (100% mortality) against *Rhyzopertha dominica* (F.) (Coleoptera: Bostrichidae), a pest of stored cereals (using a fumigant bioassay 1µL/vial of 15 mL at 30 °C in the dark), as reported in reference [7]. Another phenylpropanoid, estragole (also present in fennel EO) and fenchone were more active against *Sitophilus oryzae* L. (Coleoptera: Curculionidae) and *Callosobruchus chinensis* Fab. (Coleoptera: Bruchidae) than *(E)*-anethole [8]. *(E)*-anethole in combination with 1,8-cineole (1:1) was the best regarding fumigant toxicity on the red flour beetle adults, *Tribolium castaneum* Herbst (Coleoptera: Tenebrionidae), and it was also observed that heating enhanced the toxicity [9].

Other references in the literature [10,11,12] point out at direct contact activity of the substances against aphids and stored products pests (*Ephestia kuehniella* Zeller, Lepidoptera: Pyralidae). Fennel EO (with 67.5% of *(E)*-anethole and 25.5% of fenchone) was more active in *M. persicae* than anise (93% *(E)*-anethole), contact LD_50_ = 0.06 or 0.43%, respectively, by spraying on aphid-infested cabbage plants [13].

Solid nanoparticles of monoterpenes (carvacrol, thymol, eugenol) have been obtained using chitosan, *β*-cyclodextrin, zeine, modified starch, or polyethylen glycol (PEG) as encapsulating agents [14,15]. In previous works, beads of linalool were made by an oil emulsion entrapment method using starch, the encapsulation yield obtained was 86% and the time to release half of the bioactive exceeded 70 days [16]. Other authors prepared nanoparticles of *(E)*-anethole by emulsification and nanoprecipitation using a biodegradable polymer accepted for clinical drug delivery—polylactic-co-glycolic acid (PLGA) [17]—and after an initial burst release the activity against Gram+ bacteria lasted for more than four days. Another reference explains the encapsulation of *(E)*-anethole in liposomes, that are vesicles in which an aqueous phase is enclosed by a membrane of phospholipids; in this case, the liposomes were stable at 4 °C and provided a controlled release of *(E)*-anethole [18]. An enhancement of the antiaflatoxigenic efficacy of *I. verum* EO by nanoencapsulation in gel or lyophilized chitosan nanoparticles has also been reported [2].

Our work focusses on the use of encapsulated EOs as a fumigant system against insect pests in closed environments. For instance, solid formulations, prepared by emulsification of coriander and basil EOs in alginate and glycerol and dripping into a calcium chloride solution, were tested inside funnel traps and were as effective as the insecticide dichlorvos as killing agents for adults of the Indianmeal moth (*Plodia interpunctella* Hübner, Lepidoptera: Pyralidae) adults lured [19].

It is hypothesized that fumigant activity of plant volatiles could be exploited to control phytophagous insects of vegetables grown in greenhouses but this idea has not yet reached commercial development due to the volatility and low stability of these compounds. The objective of our work was to formulate *(E)*-anethole as solid microparticles (by oil emulsion entrapment, spray drying or molecular inclusion) and test the potential of the vapour released as aphicide on pepper leaves. Experiments were implemented with the green peach aphid, *Myzus persicae* Sulzer (Hemiptera: Aphididae), one of the main pests worldwide attacking fruit trees and vegetables and causing direct damage and transmission of virus diseases.

## 2. Results

### 2.1. Encapsulation of (E)-Anethole

The formulations prepared turned out to be within the micrometric range from 1.7 µm to 4 µm particle size. Spray drying (SD) gave the greatest encapsulation yield (73%) and loading (6.15 g/100 g of powder) of *(E)*-anethole in the capsules, although, by oil emulsion entrapment (OEE), the amount of bioactive loaded was quite similar (see Table 1).

**Figure 1:**
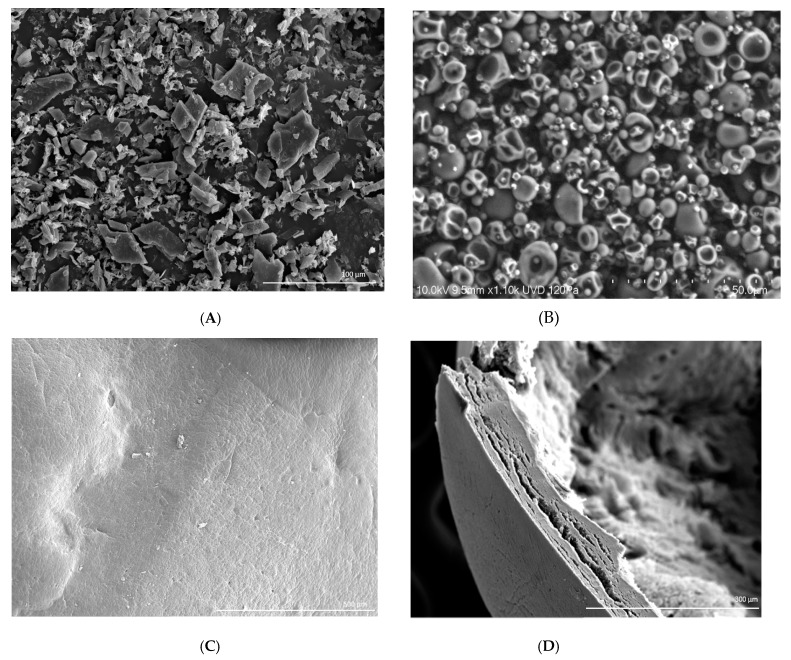
Scanning Electron Microscopy (SEM) micrographs of nanoparticles obtained by (**A**) spray drying (SD), (**B**) molecular inclusion (MI) with × 100-fold magnification, (**C**) oil emulsion entrapment (OEE) with × 100-fold magnification, and (**D**) oil emulsion entrapment (OEE) with × 190-fold magnification.

The plate in Figure 1A shows the *(E)*-anethole/β-cyclodextrin inclusion complex (MI) in the form of irregular particles, therefore this method is less suitable to encapsulate the bioactive product. On the other hand, spray drying (inlet air temperature of 100 °C) of an emulsion with maltodextrin (SD) produced spherical particles of all sizes pilled up due to the strong attraction to each other (Figure 1B). Finally, SEM micrographs (C) and (D) in Figure 1 represent the dry calcium alginate beads (OEE) and reveal a compact surface with low porosity achieved after using glycerol, the surfactant and a high percentage of sodium alginate (4%).

### 2.2. Fumigant Activity and Controlled Release of (E)-Anethole

Our formulations have good aphicidal potential (Table 2). Free *(E)*-anethole vapours were fast and entered the aphids giving the lowest LC_50_ (0.336 µL/L) after 24 h. Meanwhile OEE formulation exhibited a LC_50_ = 0.415 µL/L followed in activity by the SD preparation. Results of vapour toxicity apply just to the experimental conditions used (2.5 L dessicators plus two pepper leaves and 20 apterous *M. persicae* females in each leaf). Overall, the encapsulated *(E)*-anethole had a LC_90_ from 0.78 to 3.38 µL/L after 24 h exposure to the aphids.

**Figure 2:**
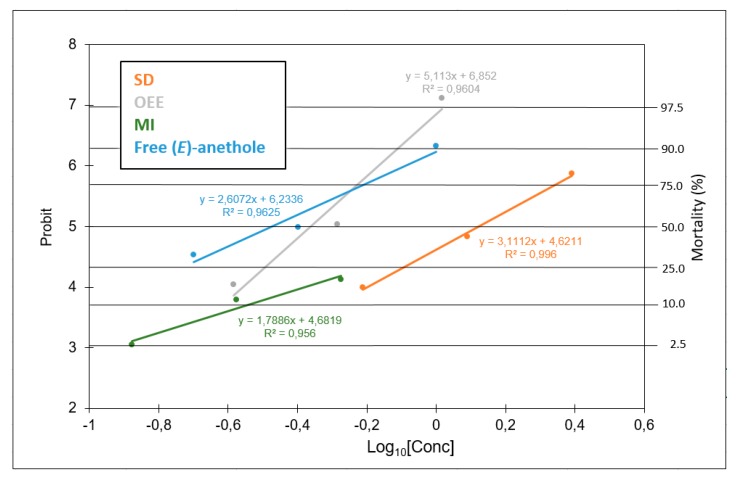
Regression lines of probit analysis for mortality against *Myzus persicae*. OEE = oil emulsion entrapment, SD = spray drying, MI = molecular inclusion, and free *(E)*-anethole.

The results are presented in more detail in Figure 2. The graph shows the dose response of the formulations including the molecular inclusion complexes (MI) for which the lethal concentrations could not be computed due to the very low mortality values obtained (this is why this treatment is not included in Table 2). The regression line of free *(E)*-anethole intercepts the probit = 5 line (that represents LC_50_) first, indicating the greatest effect at a low concentration, while the OEE formulation intercepts the probit = 6.28 (that represents LC_90_) first, indicating more effectivity at high doses (Figure 2). Overall a similar response of the preparations SD, MI, and free *(E)*-anethole is observed due to the parallel regression lines; what changes is the amount of product required to produce the same mortality.

**Figure 3:**
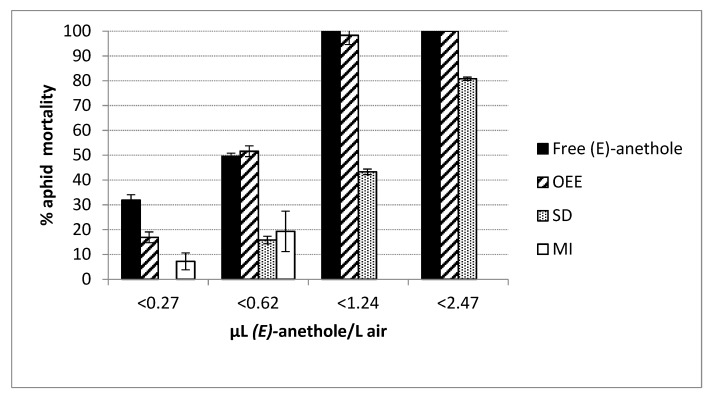
Mortality (%) in *Myzus persicae* Sulzer (Hemiptera: Aphididae) after exposure (24 h at 25 °C) to vapours (µl/L air) of *(E)*-anethole released from microspheres (OEE = oil emulsion entrapment, SD = spray drying, MI = molecular inclusion) or free *(E)*-anethole. Percentages of mortality refer to total number of insects tested in the six replications per dose and formulation (*n* = 240).

In Figure 3, we can see that the OEE formulate was quite close in toxicity to free *(E)*-anethole after 24 h, but presumably, the former would have had effects beyond the short period of observation if evaluated. In this context, MI complexes hardly produced mortality in the short term.

**Figure 4:**
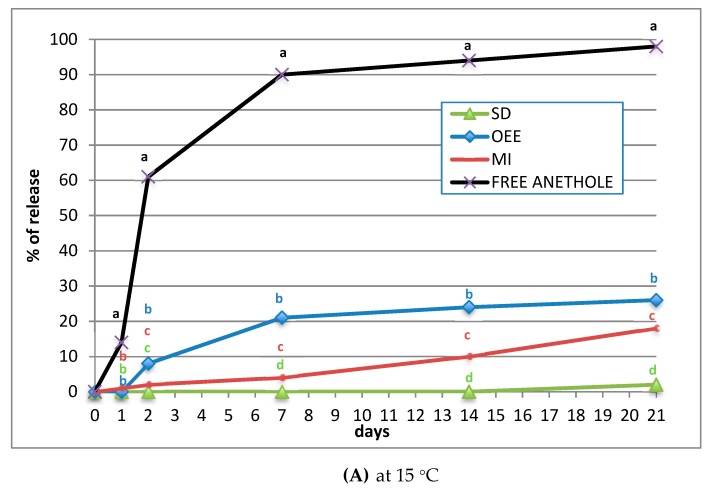

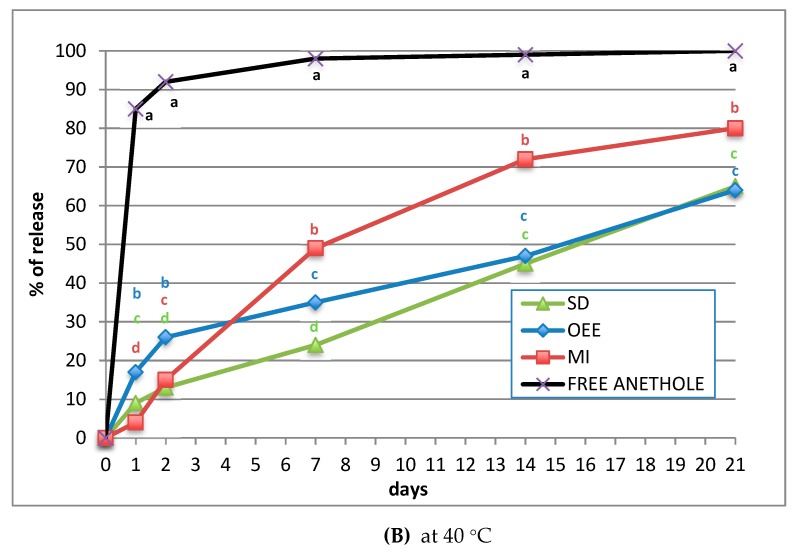
Release of free *(E)*-anethole and controlled release from formulated microspheres for 21 d (**A**) at 15 °C and (**B**) at 40 °C. Mean values in the same day with the same letter do not differ significantly (*p* > 0.05) using Duncan’s test.

Such results are explained by different paces at which *(E)*-anethole is released from the microspheres (Figure 4). At 15 °C, there are statistically significant differences among all treatments (Figure 4A), and at 40 °C, there are statistically significant differences between free *(E)*-anethole and MI but not between OEE and SD (Figure 4B). The formulation slows down the availability of the toxic vapours in comparison with the free *(E)*-anethole particularly under the conditions of the fumigant bioassay (25 °C and mortality recorded after 24 h). It is foreseen, however, that the toxic vapours would last several weeks further.

## 3. Discussion

Plants are a good natural source of *(E)*-anethole, fennel variations in the Iranian genotypes accounted for 1.2–88.4% of the EO whilst in anise 78.6–96% are common [1,20,21,22]. The mode of entry of the bioactive volatile in the insects is possibly via the respiratory system by inhalation [8,23] but its mode of action remains unclear. Some publications refer to greater activity when mixtures of volatiles for example limonene and fenchone [7,13] or 1,8-cineole [9] are applied together with *(E)*-anethole. Greater insecticidal fumigant activity against *Trichoplusia ni* Hübner (Lepidoptera: Noctuidae) of lemongrass or thyme EOs or the binary mixture of the two main compounds often had better action than pure compounds [24].

Therefore it is worthwhile to study further the fumigant effect of *(E)*-anethole in binary mixtures with monoterpenoids against *M. persicae* in all insect stages and expand the period of study (to several days) to provide new data on the advantages of a controlled release to be applied in pest control into a greenhouse.

The bioassay was done inside air-tight desiccators. Mortality was recorded after 24 h, and once opened, the concentration of the volatiles inside the desiccators could change; this was the main reason why we decided to take just one observation. Another reason was to be sure the leaves inside the desiccator were healthy enough for the aphids to remain alive, but for those affected by the insecticidal effects of anethole. The bioassay has to be improved for longer periods of observation.

If we compare our results with those of the literature, there is an agreement in the fumigant effect of EOs containing *(E)*-anethole. The lethal doses varied depending on the insect pest and the volume of the chamber used in the assays. The LD_50_ of fennel EO was 10.3 µL/L in *Brevicoryne brassicae* L. (Hemiptera: Aphidae), whereas 2 µL/L of cumin or origanum EOs (with carvacrol, *(E)*-anethole and pulegone in the oil) has been reported to cause 100% mortality in *A. gossypii* [25,26]. Our results of LC_50_ range from 0.3 to 1.47 µL/L of *(E)*-anethole (free or encapsulated) against *M. persicae*. Repellency was reported for vegetable aphid pests such as *M. persicae*, *A. gossypii*, and *Macrosiphum euphorbiae* Thomas (Hemiptera: Aphididae) in our previous works with values of RD_50_ = 0.07–0.09 µL/cm^2^ for anise and RD_50_ = 0.04–0.08 µL/cm^2^ for *(E)*-anethole [27,28], and the pure compound was more repellent for the pink clone of *M. persicae* and *A. gossypii*. The efficacy of anise EO by contact applications was greater against early nymphal instars (first and second nymphs), LD_50_ = 0.003% *v/v*, than to late nymphal instars (third and fourth nymphs), LD_50_ = 0.017% *v/v*, of apterous aphids [29]. Newly emerged adults of *T. castaneum* were highly susceptible to vapours of *(E)*-anethole in comparison with sclerotized older beetles in which concentrations at least of 20 µL/L were required to produce toxic effects [9]. Therefore, soft-bodied suckling pests such as aphids might be more susceptible to fumigation by EOs than stored product beetle pests.

Encapsulation offers clear advantages for a bioactive volatile—in addition to avoid releasing the product all at once—like protection against environmental conditions (light, temperature, oxygen, etc.). Further work will be done on improving the formulation methods described in this paper where encapsulation yields have ranged from 14 to 73%; of the three methods tested, OEE and SD are more promising. It would be of practical use the release of just the required amount of active that causes high insect mortality (previously calculated for each stage of development) for a prolonged period of time. Other authors have obtained loadings of 13%, particle size < 180 nm and bactericidal activity prolonged for more than 4 d from PLGA *(E)*-anethole nanoparticles [17]. Similarly, PEG nanoparticles of geranium and bergamot EOs slowed the release of the volatiles down and the residual contact activity against cockroaches was improved [30]. Polymer based nanoencapsulation of EO is considered for plant protection products and the type of polymers consist mainly of polysaccharides (chitosan, alginate and starch), polyesters (PEG) or biodegradable materials such as gum arabic or lecithins.

Plant essential oils are available raw materials, for example: anise EO is obtained from anise fruits at a yield of 2–6% and its market price is 7–9 €/Kg. We propose that botanicals coming from plants that have been used as foods or condiments be considered as safe plant protection products. In fact, the European Food Safety Authority (EFSA) regards them as Low Risk Active Substances (LRAS).

All classes of controlled release systems could be considered as new formulations for insecticide applications: nanocapsules or microcapsules with polymers, cyclodextrin complexes, solid-lipid nanoparticles, nanoemulsions or microemulsions, liposomes, and nanogels.

Nanotechnology is an area under development in plant protection and scaling up the experiments is important to be able to extrapolate the results to applications in agricultural production systems.

## 4. Materials and Methods

### 4.1. Materials

*(E)*-anethole (99%), calcium chloride, β-cyclodextrin (98%), maltodextrin and sodium alginate were obtained from Sigma-Aldrich, whereas glycerol (99.5% pure) was obtained from Labogros, France. Analytical grade solvents and surfactants (Tween 80) were from Sigma-Aldrich.

### 4.2. Microsphere Preparation

#### 4.2.1. Beads of (E)-Anethole by Oil-Emulsion-Entrapment (OEE)

Beads were formed by dripping an alginate solution (containing a dispersion of *(E)*-anethole, glycerol and Tween 80) into a calcium solution. Diffusion of the calcium in alginate droplets led to their gelification. The preparation of the internal phase was carried out as follows: *(E)*-anethole (20 mL) was dispersed in glycerol (20 mL) and Tween 80 (20 mL). The blend was dispersed in 20 mL of alginate (40 g/L). This dispersion was dripped into a calcium chloride solution (10 g/100 mL). Beads were filtered with a wire mesh and finally were dried overnight at room temperature (15 °C). Samples were prepared three times and then bulked.

#### 4.2.2. Preparation of β-Cyclodextrin/(E)-Anethole Molecular Inclusion (MI) Complexes

A chemical precipitation method was used to prepare β-cyclodextrin/*(E)*-anethole complexes. β-cyclodextrin (5 g) was dissolved in 300 mL of water at 55 °C for half an hour, 30 mL of *(E)*-anethole were added slowly to the suspension of β-cyclodextrin. The blend was frozen overnight at −20 °C. The precipitated *(E)*-anethole/cyclodextrin complex was recovered by lyophilization (24 h) and filtration. Samples were prepared three times and then bulked.

#### 4.2.3. Spray Drying of (E)-Anethole (SD)

An emulsion of *(E)*-anethole was mixed with 50 mL of maltodextrin (10%, *w/v*) then it was stirred at 300 rpm for 2 h. The emulsion was fed into a laboratory scale dryer (Mini Spray Dryer-B290, BÜCHI, Flawil, Switzerland) at room temperature with a flow rate of 4 mL min^−1^. The inlet and outlet temperatures were maintained at 100 °C and 60 °C, respectively. The dried powder was collected and stored in an opaque, air-tight container at 4 °C for further analysis.

### 4.3. Encapsulation Yield and Loading

The amount of *(E)*-anethole into the dry microspheres was determined by GC/MS analysis as follows: 0.5 g of powder was dispersed in 8 mL of distilled water and 4 mL of hexane in 15 mL glass vials. Vials were heated and stirred in a hot plate at 60 °C for 30 min. The organic phase containing *(E)*-anethole was decanted, and the aqueous phase was exhaustively extracted with hexane four times (4 × 4 mL). These four phases were combined. The hexane was removed using a nitrogen stream. The quantitative analysis of *(E)*-anethole was carried out using a model 5890 Series II equipped with a DB-Waters 30 m × 0.32 mm capillary column coated with a polyethylene glycol film (1 μm thickness) and an Agilent model 5972 inert mass spectrometry (MS) detector (Agilent, Palo Alto, CA, USA). The initial oven temperature was held at 60 °C for 1 min. Afterwards, it was increased by 3 °C/min to 225 °C, with injector at 250 °C, column head pressure at 8.00 psi, helium carrier gas, flow rate of 2.6 mL/min, and splitless with 2 μL of sample injected. The content of *(E)*-anethole was calculated according to the area of the chromatographic peak and using linear regression. Prior to the quantification of monoterpene, the surface *(E)*-anethole in the formulation was washed.

Encapsulation yield is defined as the ratio between the quantities of *(E)*-anethole in the capsules versus the initial amount of *(E)*-anethole. Loading is defined as the quantity of *(E)*-anethole per 100 grams of dry microcapsules. SAS version 8.0 for windows (SAS Institute, Inc., USA) was used to compare mean values of the formulations by a Tukey test at *p* < 0.05. 

### 4.4. Controlled Release of (E)-anethole through Different Matrix Blends

One gram of dry sample was placed into the vials without sealing. These vials were maintained in dry conditions at 15 °C and 40 °C in growth chambers (MLR-350H, Sanyo, Japan), and weight loss was monitored in an analytical balance as a function of time for 21 d. As a control, 1 g of *(E)*-anethole was set in a vial to study the weight loss for the same period of time. Data from three replications were recorded in this assay. Data were statistically analyzed by analysis of variance (ANOVA) using SPSS (PASW Statistic 18). Duncan’s multiple tests were applied for the calculation of the significant differences among the controlled release of the blends at the 5% level (*p* = 0.05).

### 4.5. Scanning Electron Microscopy (SEM) Analysis

Microspheres were evaluated with SEM JEOL 6100 (SAI, Universidad de Murcia, Spain). The samples were mounted (both entire structures and cross sections) on specimen stubs with double sided adhesive tape. The specimens were coated with gold and examined at an accelerating voltage of 15 kV and a working distance of 20 mm. Topographical images were collected by an image capture system used for an X-ray detector (INCA, Oxford, UK) at a magnification of 370× and 1000×. The mean particle diameter, pore diameter, number of pores per unit area and the presence of pores were recorded. SAS version 8.0 for windows (SAS Institute, Inc., Cary, NC, USA) was used to compare mean values of the formulations by a Tukey test at *p* < 0.05.

### 4.6. Fumigation Bioassay

*M. persicae*, the green peach aphid, from a laboratory culture (pink clone), maintained at a constant temperature of 25 °C and a photoperiod of 16:8 h (light:dark) on pepper plants, was used for the insecticidal experiments. The experimental unit consisted of two pepper leaves, with twenty apterous females each, placed inside a 2.5 L air-tight desiccator. Each dose was replicated six times, with 240 insects per dose. The leaf petiole was into an Eppendorf tube with water. The products, either an amount of powder of the microspheres formulations (range 0–0.1 g/L air), to obtain a dose response, or the pure free *(E)*-anethole -pipetted onto a 2.1 cm^2^ filter paper disk- (range 0–1 µl/L air), were placed in an unlid petri dish without direct contact with the insects. Therefore, only the volatile toxic effects were evaluated. The desiccators were maintained at 25 °C and 16:8 photoperiod for 24 h and aphid mortality was recorded. Controls were prepared exactly the same but the application of the products. Number of alive and dead aphids was recorded after 24 h. Probit analysis was performed to obtain LC_50_ and LC_90._

## 5. Conclusions

Spray drying of an emulsion of *(E)*-anethole with maltodextrin gave spherical particles with the greatest encapsulation yield and loading but the beads of *(E)*-anethole by oil-emulsion entrapment had better fumigant activity against *M. persicae*. Most of the free *(E)*-anethole vapours were available within 2 d of application whilst the preparations prolonged the release period for several weeks and required at least one week to release 20% of the bioactive depending on the temperature and the formulation, for instance *(E)*-anethole complexed with β-cyclodextrin required temperatures over 25 °C to release the product. Therefore, future experiments should expand the observation period and take into account the susceptibility of earlier nymphal instars to prove advantages of the practical use of *(E)*-anethole encapsulated in the form of microspheres.

## Figures and Tables

**Table 1:** Physico-chemical parameters in the microspheres.

Formulation Method ^1^	Dry Sphere Size (µm)	Loading (g Monoterpene 100 g^−1^ Powder)	Encapsulation Yield (%)
SD	4.00 a	6.15 a	73 a
OEE	1.70 b	5.20 a	26 b
MI	3.52 a	1.33 b	14 c

^1^ SD = Spray-drying, OEE = oil-emulsion-entrapment, MI = molecular inclusion (see Section 4). Samples were prepared three times and then bulked. Different letters in the same column mean significant differences at (*p* ≤ 0.05).

**Table 2:** Lethal Concentrations ^1^ of vapours of *(E)*-anethole (µl/L air) to *Myzus persicae* Sulzer (Hemiptera: Aphididae), pink clone, after 24 h.

Formulation Method ^2^	LC_50_	95% CI	LC_90_	95% CI	χ^2^
SD	1.292	1.169–1.476	3.383	2.706–4.305	0.487 ^ns^
OEE	0.415	0.416–0.468	0.780	0.675–0.832	23.850 *
Free *(E)*-anethole	0.336	0.306–0.369	1.043	0.867–1.255	8.572 ^ns^

^1^ Probit analysis fitting lethal concentration 50 (LC_50_) and 90 (LC_90_) and confidence intervals. χ^2^ non-significant (n.s.) or significant (*) at 0.1%. ^2^ SD = spray drying, OEE = oil emulsion entrapment (see Section 4).
